# Association between glucose variability and postoperative delirium in acute aortic dissection patients: an observational study

**DOI:** 10.1186/s13019-021-01456-4

**Published:** 2021-04-15

**Authors:** Yan-Juan Lin, Ling-Yu Lin, Yan-Chun Peng, Hao-Ruo Zhang, Liang-wan Chen, Xi-Zhen Huang, Qiong Chen

**Affiliations:** 1 Department of Nursing, Union Hospital, Fujian Medical University, No.29 Xinquan Road, Fuzhou, 350001 Fujian Province China; 2 Department of Nursing, Fujian Medical University, Fuzhou, China; 3 Department of Cardiac Surgery, Union Hospital, Fujian Medical University, Fuzhou, 350001 Fujian China; 4 Department of Clinical Medicine, Fujian Medical University, Fuzhou, China

**Keywords:** Glucose variability, Postoperative delirium, Acute aortic dissection patient

## Abstract

**Background:**

Blood glucose variability is associated with poor prognosis after cardiac surgery, but the relationship between glucose variability and postoperative delirium in patients with acute aortic dissection is unclear. The study aims to investigate the association of blood glucose variability with postoperative delirium in acute aortic dissection patients.

**Methods:**

We prospectively analyzed 257 patients including 103 patients with delirium. The patients were divided into two groups according to whether delirium was present. The outcome measures were postoperative delirium, the length of the Intensive Care Unit stay, and the duration of hospital stay. Multivariable Cox competing risk survival models was used to assess.

**Results:**

A total of 257 subjects were enrolled, including 103 patients with delirium. There were statistically significant differences between the two groups in body mass index, history of cardiac surgery, first admission blood glucose, white blood cell counts, Acute Physiology and Chronic Health Evaluation II score, hypoxemia, mechanical ventilation duration, and the length of Intensive Care Unit stay(*P* < 0.05). The delirium group exhibited significantly higher values of the mean of blood glucose (MBG) and the standard deviation of blood glucose (SDBG) than in the non-delirium group(*P* < 0.05). In model 1, the adjusted hazard ratio (AHR) of the standard deviation of blood glucose was 1.436(*P* < 0.05). In Model 2, the standard deviation of blood glucose (AHR = 1.418, 95%CI = 1.195–1.681, *P* < 0.05) remained significant after adjusting for confounders. The area under the curve of the SDBG was 0.763(95%CI = 0.704–0.821, *P* < 0.01). The sensitivity was 81.6%, and the specificity was 57.8%.

**Conclusions:**

Glucose variability is associated with the risk of delirium in patients after aortic dissection surgery, and high glycemic variability increases the risk of postoperative delirium.

## Background

Postoperative delirium (POD) is a severe cerebral dysfunction, and is characterized by episodes of confusion, inattention, thinking disorder and altered level of consciousness [1]. It is one of the neurological complications after cardiac surgery with an incidence of as high as 11.0–54.9% [2, 3]. And the incidence of delirium after aortic dissection surgery is 32.5–52.0% [4]. Studies have shown that POD leads to prolonged mechanical ventilation, prolonged Intensive Care Unit (ICU) stay, a 20% increased risk of long-term hospitalization for each day, the lasting delirium, and increased hospital costs [5–7]. The patients’ activity ability and quality of life are decreased were also reported [8].

In recent years, more attention has been paid to the study of blood glucose variability (GV) on disease progression and prognosis of patients, and it has important clinical significance for monitoring and controlling blood GV in severe patients [9, 10]. Compared with hyperglycemia, blood GV better reflects changes in the condition and has greater adverse effects on the body, which may have higher clinical value [10, 11]. It is well known that blood GV increases the risk of adverse events after cardiac surgery, such as acute kidney injury, and the risk of short-term and long-term death [12, 13]. At present, only one article has pointed out that hypoglycemia is positively correlated with delirium in mixed ICU diabetic patients, but high blood GV is not correlated with delirium [14]. And there is no study on the correlation between POD and blood GV in patients after cardiac surgery, nor the effect of blood GV on POD after acute aortic dissection (AAD).

According to reports, the annual incidence of AAD was 3.0–6.0 per 100,000 [4]. The mortality rate was 36–72% in AAD in the hospital during 48 h, and an increase of 1–2% in the hourly mortality rate [15]. Surgery is an important way to AAD. Therefore, this study conducted an observational study to investigate the relationship between blood GV after postoperative aortic dissection and POD, and to further clarify whether blood GV affects POD.

## Methods

### The aim and design

The study is to investigate the relationship between blood GV and POD in AAD patients who underwent surgery, and to further clarify whether blood GV affects POD, which is a prospective study.

### Participants

All patients who underwent AAD surgery at Cardiac Medical Center of Fujian Province from June 2017 to June 2019. All patients were ≥ 18 years old, no metabolic disease, no history of malignant tumor, autoimmune disease, or no severe liver and kidney dysfunction. Excluding those who stayed in ICU less than 48 h after the operation, a history of transcranial trauma, congenital deafness or schizophrenia, epilepsy before surgery, and patients who had been using glucocorticoids for a long time. Besides, patients who remained in a coma in the ICU after the operation were also excluded. The Confusion Assessment Method for the Intensive Care Unit (CAM-ICU) [16] was used to assess whether the patient had positive delirium.

The acute physiology and chronic health evaluation (APACHE-II) is the most authoritative critical illness evaluation system that has been widely used in ICU. The more serious the illness is, the higher the score is. Studies have shown that the severity of the disease is closely related to the risk of delirium [17, 18].

### Delirium assessment

Delirium was assessed by two ICU nurses who have been worked in the ICU for more than 3 years. Evaluation time was from 8:00 to 11:00, 15:00 to 17:00, and 20:00 to 23:00 on the first day after surgery, until delirium occurred or the patient transferred out of ICU.

The CAM-ICU scale can identify the following four characteristics: (1) acute onset of change or fluctuation in mental status; (2) attention disorder; (3) altered level of consciousness; (4) disorganized thinking. At the same time satisfy the features 1, 2, and 3 or 1, 2, and 4 can be a diagnosis of positive delirium.

### Data collection

After the patient was transferred to the ICU, the general demographic data, intraoperative data, and postoperative data were collected by two investigators. Blood glucose (BG) monitor was carried out every 2–6 h according to the glucose control after the doctors evaluated the patient’s condition. We started to collect the BG values obtaining from the arterial blood gas analysis at 8 am on day one postoperatively, which continuously for 48 h. If the interval of two blood glucose monitors was longer than 6 h, the patient was excluded. Mean blood glucose (MBG) level as an arithmetic mean of all recorded glucose values for each patient for 48 h, and the variability of blood glucose was assessed by the standard deviation of blood glucose (SDBG).

### Power and sample size

The sample size of this study was calculated using the Leslie Kish formula [19] for sample size determination for a single proportion as follows: $n={Z}_{a/2}^2\pi \left(1-\uppi \right)/{\delta}^2$. According to one review, the prevalence of delirium after aortic dissection surgery is 32.5–52.0% [4], which we used the prevalence of 52% in order to obtain the maximum possible sample size that provided more precise estimates. Considering the existence of shedding and other factors, the sample size was enlarged by 20%. Thus, the final calculated sample size is 115 patients.

### Statistical analysis

We used Excel (Microsoft Corporation, Redmond, WA, USA) to calculate the SDBG and the MBG, and SPSS statistical package of 21.0 version for statistical analysis, using the appropriate statistical methods to describe data. The continuity variable conforms with the normal distribution uses the t-test and the non-compliance with the normal distribution uses the Wilcoxon signed rank-sum test. The length of ICU stay was the time index, calculating in days. It started from the first day when patients stay in the ICU until the ICU discharge. Multivariate Cox regression analysis was performed to determine whether delirium occurred as a dependent variable (event), and hazard ratio (HR) values and 95% confidence intervals (CI) were obtained. *P* < 0.05 was considered statistically significant.

## Results

**Fig. 1:**
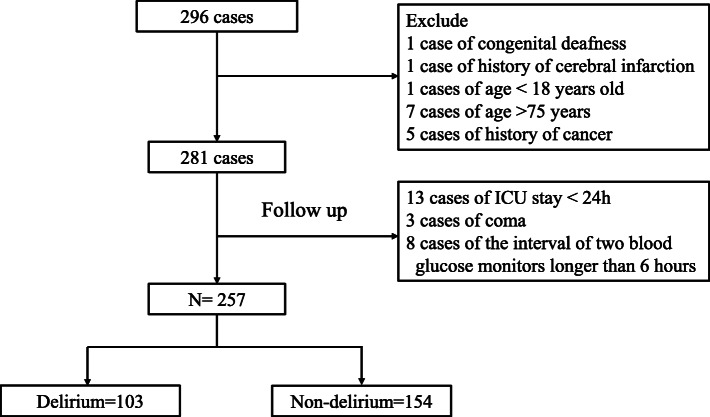
Patients' flowchart of the study

From June 2017 to June 2019, 296 patients with AAD were included, and 257 patients were finally included, as shown in Fig. 1. There were 103 patients with delirium and 154 patients without delirium, and the incidence of delirium was 40.08%.

Table 1 shows the demographic and clinical characteristics of the delirium group and the non-delirium group. There was no statistically significant difference in age between the two groups(*P* > 0.05). There were statistically significant differences between the two groups in body mass index (BMI), history of cardiac surgery, first admission blood glucose, white blood cell (WBC) count, APACHE-II score, hypoxemia, mechanical ventilation duration, and the length of ICU stay(*P* < 0.05). The delirium group exhibited significantly higher values of MBG and SDBG than in the non-delirium group(*P* < 0.05).

**Table 1:** Characteristics of delirium patients and non-delirium patients

Variables	Delirium (*n* = 103)	Non-delirium (*n* = 154)	*P*
**Demographic information**			
Age, mean (SD), years	53.3 ± 10.5	51.1 ± 12.8	0.145
BMI, median IQR, kg/m^2^	25.4 (23.5, 27.7)	24.0 (22.2, 26.7)	0.014
Male, n (%)	81 (78.6)	113 (73.4)	0.336
Smoker, n (%)	41 (39.8)	60 (39.0)	0.892
Drinker, n (%)	41 (39.8)	56 (36.4)	0.577
High school and above, n (%)	20 (19.4)	36 (23.4)	0.374
Married, n (%)	100 (97.1)	152 (98.7)	0.648
Hypertension, n (%)	83 (80.6)	112 (72.7)	0.149
History of cardiac surgery, n (%)	7 (6.8)	2 (1.3)	0.019
**Preoperative data**			
First admission blood glucose, IQR, mmol/L	7.6 (6.6, 9.2)	6.9 (5.9, 7.8)	< 0.001
WBC, mean (SD), [×10^9^/L]	12.9 ± 4.1	11.8 ± 3.9	0.033
Neutrophil, median (IQR), [×10^9^/L]	10.0 (7.0, 12.6)	8.5 (4.6, 11.5)	0.061
Lymphocyte, median (IQR), [×10^9^/L]	6.5 (4.1, 10.0)	7.1 (4.1, 11.3)	0.330
Monocyte, median (IQR), [×10^9^/L]	5.0 (3.5, 8.0)	5.4 (2.6, 7.6)	0.344
RBC, median (IQR), [×10^9^/L]	4.3 (3.9, 4.8)	4.3 (4.0, 4.7)	0.922
Platelet, median (IQR), [×10^9^/L]	196.0 (149.8, 235.0)	193.0 (149.8, 217.0)	0.138
Hb, median (IQR), g/L	134.0 (122.0, 146.0)	131.0 (118.0, 142.0)	0.175
Anemia, n (%)	18 (17.5)	21 (13.6)	0.896
ASA grade, n (%)			0.608
III level	20 (19.4)	34 (22.1)	
≥ IV level	83 (80.6)	120 (77.9)	
Entry status, n (%)			0.173
Quiet	50 (48.5)	64 (41.6)	
nervous	33 (32.0)	67 (43.5)	
confused	20 (19.4)	23 (14.9)	
**Intraoperative data**			
Operating time, median (IQR), minutes	299.0 (255.0, 365.0)	290.5 (253.5, 362.5)	0.642
Aortic cross-clamp time, median (IQR), minutes	65.0 (46.0, 95.0)	57.0 (43.0, 95.0)	0.326
CPB, median (IQR), minutes	155.0 (130.0, 188.0)	149.0 (125.0, 186.5)	0.600
Blood loss, median (IQR), ml	800.0 (600.0, 1000.0)	800.0 (600.0, 1000.0)	0.367
**Postoperative data**			
APACHE-II scores, n (%)			< 0.001
< 15	38 (36.9)	111 (72.1)	
15–20	46 (44.7)	32 (20.8)	
> 20	19 (18.4)	11 (7.1)	
Hypoxemia, n (%)	52 (50.5)	25 (16.2)	< 0.001
MBG, mmol	13.1 (11.6, 15.0)	10.7 (9.6, 11.9)	0.001
SDBG, mmol	2.9 (2.2, 3.9)	1.7 (1.3, 2.5)	< 0.001
ICU stay, median (IQR), day	7.0 (5.0, 10.0)	5.0 (4.0, 7.0)	< 0.001
Mechanical ventilation duration, median (IQR), hours	61.0 (37.0, 139.0)	42.5 (27.8, 82.3)	< 0.001
Hospitalization days, median (IQR), day	21.0 (15.0, 26.0)	18.0 (14.0, 25.0)	0.357

Values are n (%) unless otherwise indicated

*SD* Standard Deviation, *BMI* Body Mass Index, *IQR* Interquartile, *WBC* White Blood Cell, *RBC* Red Blood Cell, *Hb* Hemoglobin, *ASA* American Society of Anesthesiologists, *CPB* Cardiopulmonary Bypass, *APACHE* Acute Physiology and Chronic Health Evaluation Score, *MBG* Mean of Blood Glucose, *SDBG* Standard Deviation of Blood Glucose, *ICU* Intensive Care Unit

In Table 2, patients are divided into lower GV group and higher GV group using the median cut-off of the standard deviation of blood glucose. There were no statistically significant differences in age, BMI, gender, smoker, drinker, education level, marital status, hypertension, and history of cardiac surgery, etc. Compared with the lower GV group, the length of ICU stay in the higher GV was longer, and the difference between the two groups was statistically significant(*P* < 0.05).

**Table 2:** Baseline characteristics of patients with low and high GV

Variables	Lower GV *N* = 129	Higher GV *N* = 128	*P*
Age, mean (SD), years	50.8 ± 12.7	53.1 ± 11.0	0.117
BMI, median IQR, kg/m^2^	24.8 (22.8, 27.2)	24.2 (22.5, 26.7)	0.575
Male, n (%)	97 (75.2)	97 (75.8)	0.913
Smoker, n (%)	52 (40.3)	45 (35.2)	0.394
Drinker, n (%)	53 (41.1)	48 (37.5)	0.556
High school and above, n (%)	31 (24.0)	25 (19.5)	0.190
Hypertension, n (%)	92 (71.3)	103 (80.5)	0.086
Married, n (%)	127 (98.4)	125 (97.7)	0.645
History of cardiac surgery, n (%)	2 (1.6)	7 (5.5)	0.088
First time blood glucose, median (IQR), mmol	7.0 (5.9, 8.5)	7.2 (6.2, 8.3)	0.274
WBC, median (IQR), [×10^9^/L]	12.4 ± 3.8	12.2 ± 4.2	0.643
Neutrophil, median (IQR), [× 10^9^/L]	9.2 (5.7, 11.7)	9.0 (5.5, 12.3)	0.880
Lymphocyte, median (IQR), [×10^9^/L]	6.7 (3.7, 10.4)	7.2 (4.5, 11.5)	0.202
Monocyte, median (IQR), [×10^9^/L]	4.8 (2.2, 7.7)	5.8 (3.8, 7.9)	0.085
RBC, median (IQR), [×10^9^/L]	4.3 (4.0, 4.7)	4.4 (3.9, 4.7)	0.909
Platelet, median (IQR), [×10^9^/L]	192.0 (150.0, 234.5)	187.5 (149.3, 225.8)	0.846
Hb, median (IQR), g/L	131.0 (119.5, 141.5)	134.0 (121.0, 145.0)	0.251
Anemia, n (%)	21 (16.3)	18 (14.1)	0.620
Mechanical ventilation duration, median (IQR), hours	45.0 (33.5, 96.0)	48.3 (28.3, 102.2)	0.609
ICU stay, median (IQR), day	6.0 (4.0, 8.0)	6.0 (4.0, 10.0)	0.040
Hospitalization days, median (IQR), day	19.5 (14.3, 25.0)	20.0 (15.0, 27.0)	0.455

Values are n (%) unless otherwise indicated

*SD* Standard Deviation, *IQR* Interquartile Range, *BMI* Body Mass Index, *GV* Glucose Variability, *WBC* White Blood Cell, *RBC* Red Blood Cell, *Hb* Hemoglobin, *ICU* Intensive Care Unit

As shown in Table 3, after adjusting for age, gender, and BMI in model 1, the adjusted hazard ratio (AHR) of WBC was 0.932(*P* > 0.05), which was not correlated with the risk of POD; the AHR of APACHE-II > 20 scores was 2.178(95% CI = 1.108–4.281), the AHR of hypoxemia was 1.563(95% CI = 1.070–2.518), and the AHR of SDBG was 1.436(95% CI = 1.205–1.711), all three increased the risk of delirium(*P* < 0.05). In Model 2, the AHR of APACHE-II > 20 scores was 2.376(95% CI = 1.342–3.876), the AHR of hypoxemia was 1.778(95% CI = 1.122–2.818), and the AHR of SDBG was 1.418(95% CI = 1.195–1.681), all three remained significant after adjusting for confounding factors(*P* < 0.05).

**Table 3:** Multivariable Cox regression analysis of possible predictors of delirium

	Model 1		Model 2	
Variables	AHR(95% CIs)	*P*	AHR(95%CIs)	*P*
Age, years	1.007 (0.985–1.028)	0.547	1.002 (0.983–1.002)	0.813
BMI, kg/m^2^	1.029 (0.971–1.091)	0.336	1.021 (0.963–1.083)	0.482
Male	1.381 (0.801–2.381)	0.246	–	–
WBC	0.932 (0.852–1.020)	0.124	0.993 (0.994–1.046)	0.802
First time blood glucose	1.012 (0.968–1.058)	0.607	1.015 (0.973–1.058)	0.931
Neutrophil	1.062 (0.988–1.143)	0.105	–	–
APACHE-II (> 20 scores)	2.178 (1.108–4.281)	0.024	2.376 (1.342–3.876)	0.002
Hypoxemia	1.563 (1.070–2.518)	0.046	1.778 (1.122–2.818)	0.014
SDBG, mmol	1.436 (1.205–1.711)	< 0.001	1.418 (1.195–1.681)	0.001
Mechanical ventilation duration	1.000 (0.999–1.002)	0.791	1.000 (0.998–1.001)	0.931

*BMI* Body Mass Index, *WBC* White Blood Cell, *APACHE* Acute Physiology and Chronic Health Evaluation Score, *SDBG* Standard Deviation of Blood Glucose

**Fig. 2:**
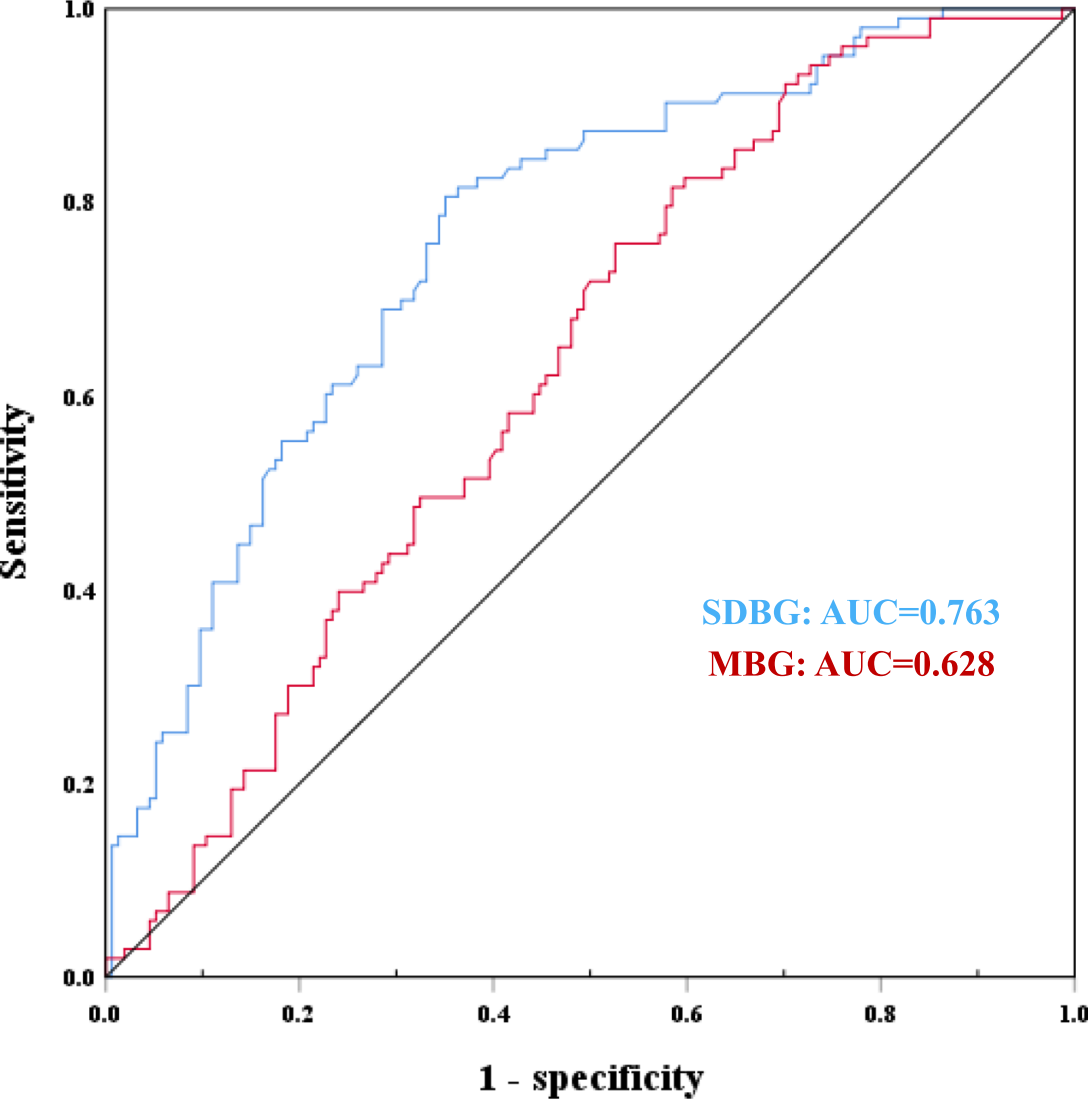
The ROC curves of predict POD of patients with AAD. The area under the curve of the SDBG was 0.763(*P* < 0.001). The sensitivity was 81.6%, and the specificity was 57.8%. The area under the curve of the MBG was 0.628(*P* = 0.001). The sensitivity was 75.7%, and the specificity was 53.2%

According to the receiver operating characteristic (ROC) curve, the area under the curve of the SDBG was 0.763(*P* < 0.01). The sensitivity was 81.6%, and the specificity was 57.8%. The area under the curve of the MBG was 0.628(*P* = 0.001). The sensitivity was 75.7%, and the specificity was 53.2%. The difference of area under the curve between the two groups was statistically significant(*P* = 0.002), which was shown in Fig. 2.

## Discussion

Delirium is a common complication after aortic dissection surgery with an incidence of 32.5 to 52.0% [4], and the results of this study showed that the incidence of delirium was 40.08%, which was consistent with other studies. The area under the curve of the SDBG was greater than the MBG. After adjusting for confounding factors, the SDBG was independently correlated with the risk of delirium.

No matter how the quality of perioperative blood glucose controls in patients who underwent cardiac surgery, blood glucose changes are reported they are related to postoperative complications [20, 21]. Hypoglycemia and hyperglycemia have been identified as risk factors for delirium [22, 23], but there are few reports on the relationship between glucose fluctuation and delirium. Keulen et al. [14] shows that delirium is positively associated with hypoglycemia in severe patients with diabetes, but not associated with pronounced glycemic variability. And Heymann et al. [24] found that patients with hyperactive delirium have higher MBG than the non-hyperactive delirium patients. There is no consensus on the relationship between glucose fluctuation and delirium, and this study showed that high glycemic variability increases the risk of postoperative delirium.

Glucose, a simple carbohydrate, is the main source of energy for many cells. Studies have shown that the brain consumes 50% of the total body’s consumption of glucose [25]. Glucose sensory neurons are present in several areas of the brain. The activity of neurons changes with the level of glucose, and the brain function depends on the stable glucose levels, which is why the brain is particularly sensitive to glucose level. Therefore, the blood glucose level needs to be maintained in a narrow physiological range [26]. A study shows that people with diabetes are at least 1.5 times more likely to develop dementia than people without diabetes, further highlighting the effect of glucose changes on brain function and long-term consequences [25]. Both acute and chronic hyperglycemia has been shown to cause oxidative stress, subsequent neuronal damage, and cognitive decline [27], and the reason is closely related to delirium.

At present, the mechanism of blood glucose volatility promoting the development of critical illness and poor prognosis is not clear. However, blood glucose volatility is the biological basis of the human body [28]. According to the theory of oxidative stress, fluctuating hyperglycemia can easily cause oxidative stress than persistent hyperglycemia [29]. The specific mechanism may be that intermittent hyperglycemia can increase the overexpression of reactive oxygen species in the mitochondrial transport chain, thereby promoting oxidative stress response, increasing the apoptosis rate of endothelial cells, and ultimately causing damage to central nervous function [27]. Meanwhile, it has been reported that the oxidative stress of intermittent hyperglycemia is greater than that of sustained hyperglycemia under experimental conditions, which has been confirmed by clinical studies. Besides, high blood glucose will also cause the release of a large number of pro-inflammatory cytokines, resulting in coagulation dysfunction, vascular reactivity abnormalities, and other injuries. However, patients with acute aortic dissection often present intermittent hyperglycemia due to acute illness, surgical stress, drugs, and other reasons, which are closely involved in a central nervous injury.

Cardiopulmonary bypass is the main technology for acute aortic dissection. Inflammatory mediators are released in large quantities during cardiopulmonary bypass cardiac surgery, and the inflammatory state is another common phenomenon of a stress response. The inflammatory response itself has a protective effect on the body. But when the balance between inflammatory and anti-inflammatory is broken, the body shows an inflammatory state and a large number of inflammatory factors in the peripheral circulation enter the central nervous system through various channels, which can cause inflammation in the central nervous system [30]. A large number of studies have suggested that neuron inflammation may be one of the mechanisms of cognitive impairment. Cibelli et al. [31] founds that surgical trauma activates the innate immune system, which in turn triggers an IL-1-mediated inflammatory response in the hippocampus, resulting in memory impairment in mice. Besides, the operation itself is a kind of serious trauma, causing the organism to appear stress state. The imbalance of the central nervous system about noradrenaline and acetylcholine decreases the acetylcholine content, resulting in a series of neurological complications.

There are several limitations to this study. First, the sample size was small, and the relevant conclusions need to be further demonstrated with a larger sample. Second, we only evaluated delirium in ICU patients without long-term follow-up. Finally, continuous glucose monitoring was used to continuously measure blood glucose in recent studies, which the frequency of BG tests in our study may not be enough. In future studies, we will use the continuous glucose monitoring for real-time monitoring of patients.

## Conclusions

Glucose variability is associated with the risk of delirium in patients after aortic dissection surgery, and high glycemic variability increases the risk of POD. Therefore, we should pay more attention to it.

## Data Availability

The datasets used and analyzed during the current study are available from the corresponding author on reasonable request.

## Abbreviations

POD: Postoperative delirium
GV: Glucose variability
ICU: Intensive Care Unit
AAD: Acute aortic dissection
MBG: Mean of blood glucose
SDBG: Standard deviation of blood glucose
SD: Standard deviation
CAM-ICU: The Confusion Assessment Method for the Intensive Care Unit
BG: Blood glucose
AHR: Adjusted hazard ratio
HR: Hazard ratio
CI: Confidence intervals
BMI: Body mass index
WBC: White blood cell
APACHE-II: Acute physiology and chronic health evaluation score
ROC: Receiver operating characteristic
IQR: Interquartile
RBC: Red blood cell
Hb: Hemoglobin
ASA: American Society of Anesthesiologists
CPB: Cardiopulmonary bypass
